# Estimating fetal exposure to the P‐gp substrates, corticosteroids, by PBPK modeling to inform prevention of neonatal respiratory distress syndrome

**DOI:** 10.1002/psp4.12674

**Published:** 2021-07-23

**Authors:** Olena Anoshchenko, Mark A. Milad, Jashvant D. Unadkat

**Affiliations:** ^1^ Department of Pharmaceutics School of Pharmacy University of Washington Washington Seattle USA; ^2^ Milad Pharmaceutical Consulting LLC Plymouth Michigan USA

## Abstract

We have previously developed a maternal‐fetal physiologically‐based pharmacokinetic (m‐f PBPK) model to dynamically predict (and verify) fetal‐maternal exposure to drugs that passively diffuse across the placenta. Here, we extended the application of this model to dynamically predict fetal exposure to drugs which are effluxed by placental P‐glycoprotein, namely the antenatal corticosteroids (ACS; dexamethasone [DEX], and betamethasone [BET]). To do so, we estimated both the placental P‐gp mediated efflux clearance (CL) and the passive diffusion CL of the ACS. The efficacy and toxicity of the currently used maternal ACS dosing regimens to prevent neonatal respiratory distress syndrome could be improved by altering their dosing regimens. Therefore, to illustrate the utility of our m‐f PBPK model, we used it to design alternative dosing regimens of DEX and BET that could potentially improve their efficacy and reduce their toxicity. The redesigned dosing regimens are convenient to administer, maintain maternal‐fetal exposure (area under the concentration‐time curve [AUC]) or maximum plasma concentration (C_max_) or both (DEX and BET) or minimize maternal exposure while maintaining fetal drug plasma concentrations above the minimum therapeutic threshold of 1 ng/ml for 48 h (BET only; based on efficacy data in sheep). To our knowledge, this is the first study to dynamically predict fetal plasma concentrations of placental P‐gp effluxed drugs. Our approach and our m‐f PBPK model could be used in the future to predict maternal‐fetal exposure to any drug and to design alternative dosing regimens of the drug.

**Study Highlights**

**WHAT IS THE CURRENT KNOWLEDGE ON THE TOPIC?**

Fetal exposure to drugs is logistically and ethically challenging to measure, even at the time of birth when umbilical and maternal venous samples can be obtained. We previously developed a maternal‐fetal physiologically‐based pharmacokinetic (m‐f PBPK) model to dynamically predict (and verify) fetal:maternal exposure to drugs that passively diffuse across the placenta. However, this model has never been applied to drugs that are transported by placental transporters.

**WHAT QUESTION DID THIS STUDY ADDRESS?**

Here, we extended our m‐f PBPK model to dynamically predict fetal exposure to drugs which are effluxed by placental P‐glycoprotein, namely the antenatal corticosteroids.

**WHAT DOES THIS STUDY ADD TO OUR KNOWLEDGE?**

To our knowledge, this is the first study to dynamically predict fetal plasma concentrations of placental transported drugs.

**HOW MIGHT THIS CHANGE CLINICAL PHARMACOLOGY OR TRANSLATIONAL SCIENCE?**

Our approach and our m‐f PBPK model could be used in the future to predict maternal‐fetal exposure to any drug (including those that are transported) and to devise dosing regimens of the drug to maximize maternal‐fetal drug efficacy and minimize drug toxicity.

## INTRODUCTION

Approximately 80% of pregnant women take prescription or over‐the‐counter medications^1^ to treat a variety of maternal conditions (e.g., diabetes) or to treat the fetus (e.g., prevent respiratory distress syndrome [RDS]). To maximize maternal‐fetal drug efficacy and minimize toxicity, it is critical that fetal exposure to the drug be quantified. Such quantification is logistically and ethically challenging. At the most, a single blood sample from the umbilical vein (UV) and maternal vein (MP) can be obtained at the time of birth. The ratio of the plasma drug concentration in these samples (UV/MP) is not a measure of fetal exposure as it only provides a snapshot of the fetal:maternal drug concentration at a single time point.^2^ If such data are available over multiple time points, covering several half‐lives of the drug, the data can be “naively” pooled to arrive at a complete UV/MP concentration‐time profile. From this profile can be derived *K_p,uu_*, the steady‐state unbound fetal:maternal partition coefficient of the drug. When a drug passively diffuses across the placenta and provided there is minimal placental or fetal clearance of the drug, *K_p,uu_* = 1. However, many drugs administered to pregnant women are substrates (e.g., antenatal corticosteroids used to prevent RDS) of transporters that are highly abundant in the placenta (e.g., P‐glycoprotein [P‐gp]). When a drug is a substrate of placental efflux transporters, *K_p,uu_* < 1. While *K_p,uu_* provides an estimate of the fetal exposure to the drug at steady‐state, it cannot be used to dynamically predict fetal plasma concentration time profile. To do so, one needs the actual values of the transplacental clearances. Provided there is minimal placental and fetal elimination of the drug, K_p,uu_ is related to these transplacental clearance (CL) as follows:

(1) $$K_{p,uu}=\frac{{\text{CL}}_{\text{int,PD,placenta}}}{{\text{CL}}_{\text{int,PD,placenta}}+{\text{CL}}_{\text{int,Pgp,placenta}}}$$

Where CL_int,PD,placenta_ and CL_int,Pgp,placenta_ are the in vivo intrinsic placental passive diffusion clearance and P‐gp mediated efflux clearance, respectively.

We have previously developed and verified a maternal‐fetal physiologically‐based pharmacokinetic (m‐f PBPK) model to dynamically predict fetal:maternal exposure to drugs that passively diffuse across the placenta.^2^, ^3^ To do so, we developed an innovative approach to estimate the transplacental CL of the drugs (based on midazolam as model placental passive diffusion drug). Our m‐f PBPK model incorporates all the changes in gestational‐age dependent physiological parameters that are important in drug disposition. For example, this model includes gestational‐age dependent changes in cardiac output, organ blood flows (including to the placenta), total body water, plasma protein concentrations (e.g., albumin), changes in hepatic CYP activity, placenta size, fetal growth, etc.^2^, ^3^ Thus, our model can predict fetal exposure to drugs at any gestational age. However, even though this model is capable of incorporating placental drug transport, it has never been used to dynamically predict fetal exposure to drugs that are transported by the placenta. Here, using antenatal corticosteroids (ACS) as an example, we extend this model to do so.

ACS, dexamethasone (DEX), and betamethasone (BET) were chosen as our model drugs because they are the most common ACS used to prevent RDS and are substrates of P‐gp. These two epimers were developed in the early 1960s to treat rheumatoid arthritis; however, the efficacy (prevention of RDS) and toxicity (e.g., maternal infection,^4^, ^5^ fetal neurodevelopmental disorders,^6^, ^7^ and hypoglycemia^4^) of ACS has not been systematically addressed.^5^ To prevent RDS, the usual ACS dosing regimens administered to pregnant women of 24 to 36 gestational weeks (GWs) are intramuscular (i.m.) administration of 6 mg DEX phosphate (DEX‐P) every 12 h for 48 h, or 12 mg of 1:1 BET phosphate:acetate mixture (BET‐P:A) every 24 h for 48 h^8^, ^9^ (henceforth referred to as the reference dosing regimens). The efficacy of these reference regimens is modest. For example, a recent large clinical trial in low to middle income countries found that the risk of both neonatal death and RDS in preterm babies was reduced by only one‐sixth when the DEX reference regimen was administered to pregnant women at imminent risk of preterm birth (within 48 h).^5^ Alternatively stated, despite ACS therapy, the relative risk of RDS or infant mortality within 28 days of birth remains high (relative risk of RDS of 0.81 and 0.84, respectively). Collectively, these data suggest a need to further refine DEX and BET dosing regimens to prevent RDS.

ACS safety and efficacy are linked to their fetal (and maternal) drug exposure, but defining this relationship is difficult. First, as indicated above, UV/MP drug concentration at a single time point does not provide a measure of fetal exposure to ACS. Second, maternal drug exposure is not a good surrogate of fetal exposure as these drugs are substrates of P‐gp^10^, ^11^ and acts to variably diminish fetal ACS exposure relative to that in the mother.^12^, ^13^, ^14^ The extent of this diminution and the variability therein has not been well‐defined, but likely changes as pregnancy progresses due to the gestational‐age dependent change in placental P‐gp protein abundance.^15^ Finally, even though DEX and BET are epimers, they differ in their pharmacokinetic characteristics. For example, BET has lower hepatic clearance and longer half‐life than DEX.^16^, ^17^ In addition, the formulation of the two ACS used for intramuscular administration differ, resulting in different pharmacokinetics. BET is administered as the 1:1 mixture of the phosphate (BET‐P; fast release) and the acetate (BET‐A; slow release) resulting in a sustained release of BET from the i.m. depo site compared to the rapid release of DEX following DEX‐P i.m. administration.^18^

**FIGURE 1:**
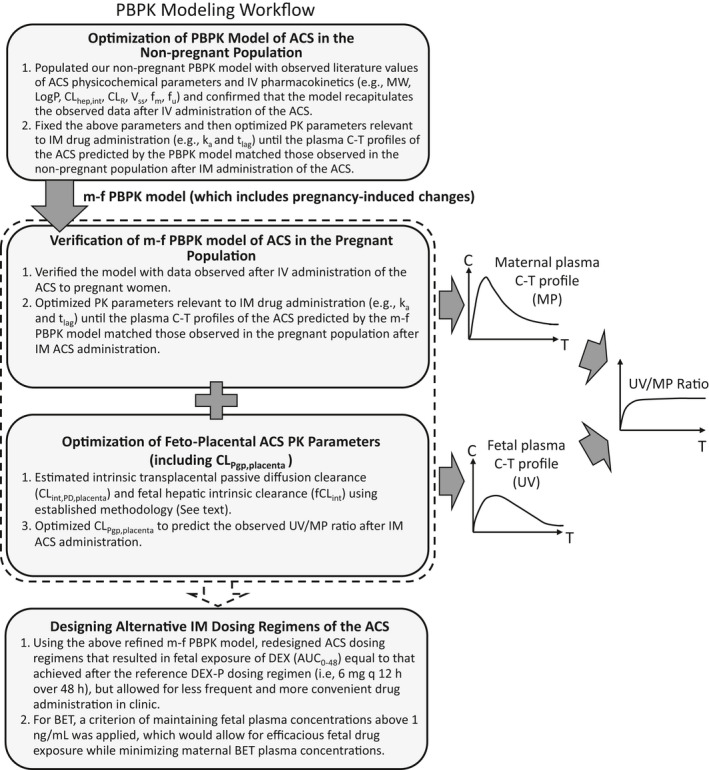
General workflow of PBPK modeling and simulation. Of note, the m‐f PBPK model used was exactly the same as previously described in our two publications by Zhang et al., 2017 (see Introduction and Methods). This m‐f PBPK model already incorporates placental P‐gp efflux clearance functionality. Because the antenatal corticosteroids (ACS) are P‐gp substrates, we utilized this functionality to dynamically predict fetal exposure to the ACS. AUC, area under the concentration‐time curve; BET, betamethasone; CL, clearance; C‐T, concentration‐time; DEX, dexamethasone; m‐f PBPK, maternal‐fetal physiologically‐based pharmacokinetic; MP, maternal vein; PBPK, physiologically‐based pharmacokinetic; PK, pharmacokinetic; UV, umbilical vein

To overcome the aforementioned challenges in determining fetal ACS exposure, methods to accurately predict (rather than measure) are needed. Therefore, we applied our recently developed m‐f PBPK model to dynamically predict fetal exposure to the ACS. To do so, we needed to estimate both the CL_int,Pgp,placenta_ and CL_int,PD,placenta_ of the ACS (see Figure 1 for workflow). The latter was estimated using our previously adopted innovative approach that is based on the passive diffusion CL of midazolam (a model passive diffusion drug). The former was based on the in vivo *K_p,uu_* of the ACS estimated using the observed UV/MP values obtained from multiple maternal‐fetal dyads. That is, we optimized the magnitude of placental P‐gp efflux clearance (CL_int,Pgp,placenta_) of each ACS to match (as closely as possible) the observed UV/MP ratios.

Because maternal plasma drug concentrations drive fetal plasma drug concentrations, to accomplish the above goals, we first optimized ACS plasma concentration in the non‐pregnant population (top panel, Figure 1). Second, without changing any of the pharmacokinetic (PK) parameters of the ACS, they were input into our m‐f PBPK model. This model incorporates all the physiological changes caused by pregnancy (including the 2‐fold induction in hepatic CYP3A activity). Only the *k_a_* and *t*_lag_ of the ACS were optimized to match the observed plasma concentration‐time (C‐T) profile of the ACS in pregnant women (second panel of Figure 1). Then, the fetal‐placental ACS PK parameters were estimated (CL_int,PD,placenta_) or optimized (CL_Pgp,placenta_) to match the observed UV/MP ratio after i.m. administration of the ACS (third panel of Figure 1). Finally, to illustrate the utility of our approach, all the above ACS maternal‐fetal PK parameters were input into our m‐f PBPK model to dynamically simulate fetal exposure to several ACS dosing regimens alternative to those used in the clinic.^19^ These alternative dosing regimens have the potential to increase the efficacy and reduce the toxicity of the ACS.

## MATERIALS AND METHODS

Please, see Supplementary Information.

## RESULTS

### Verification of Simcyp PBPK model of ACS using the observed data from the non‐pregnant Indian population

**FIGURE 2:**
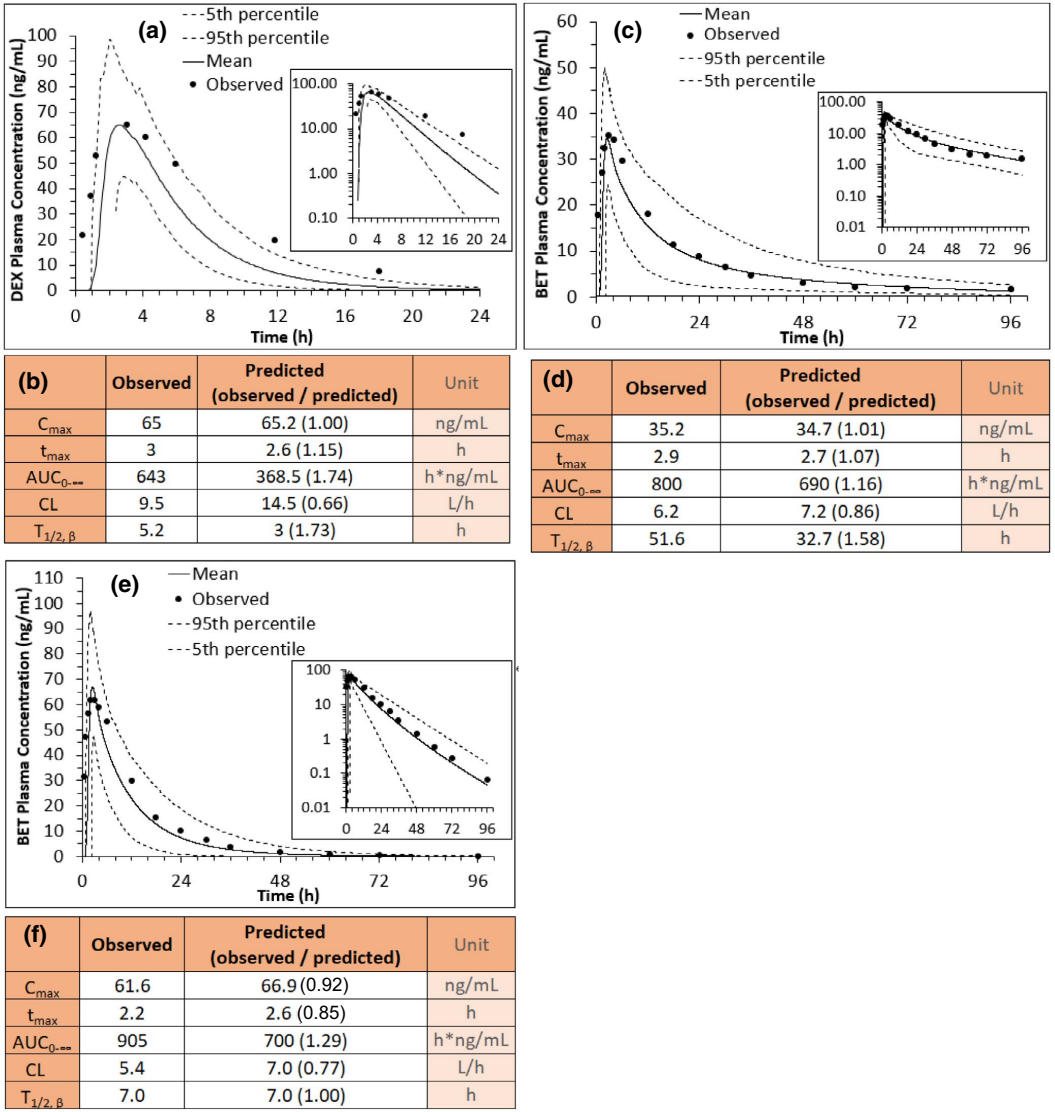
Verification of model predicted plasma concentration‐time profile after i.m. betamethasone (BET) or dexamethasone (DEX) administration to Indian (Bangalore) non‐pregnant women (a) 6 mg DEX phosphate (c) 6 mg BET phosphate:acetate mixture (e) 6 mg BET phosphate. Model predicted mean values and their 5th and 95th percentiles are solid and dashed lines respectively (b, d, f). Comparison of the observed and predicted pharmacokinetic parameters of profiles shown in a, b, and c, respectively showed that model predicted plasma concentration‐time profiles were verified for BET‐P:A (d) but not for DEX‐P (b) or BET‐P (f). BET profiles in c were generated using dual absorption input function, where half of the dose (phosphate) was absorbed from the i.m. site with *k_a_*_1_ = 1.5 h^−1^ and the other half (acetate) was absorbed with *k_a_*_2_ = 0.2 h^−2^. Observed PK parameters were reported previously by Jobe et al., 2020 or estimated from the digitized mean concentration‐time profiles using noncompartmental analysis using Phoenix 8.1 (linear trapezoid method was employed). Insets show the ACS concentrations plotted on a log scale. AUC, area under the concentration‐time curve; CL, clearance; C_max_, maximum plasma concentration; T_1/2_, terminal half‐life; T_max_, time to maximum plasma concentration

When the SimCYP Simulator was populated with observed clearance, volume of distribution at steady state (V_ss_), renal clearance (CL_R_), and other PK parameters obtained in the non‐pregnant White population after i.v. administration of DEX and BET, the model recapitulated the observed ACS plasma concentration‐time profiles within the a priori defined acceptance criteria (data not shown). Then, when the model, populated with these parameters, was used to predict area under the concentration‐time curve from zero to infinity (AUC_0–∞_) and CL in the Indian population after BET‐P:A i.m. administration, the predicted values fell within 0.8‐ to 1.25‐fold of the observed values (except terminal half‐life [T_1/2,β_]; Figure 2c,d). In contrast, based on our acceptance criteria, after DEX‐P (Figure 2a,b) and BET‐P i.m. administration (Figure 2e,f), the model underpredicted the AUC_0–∞_ and overpredicted CL observed in the Indian population. The BET‐P:A (i.m.) data in the Indian non‐pregnant population were used for verification ONLY because the corresponding data in the White population are not available.

### Verification of m‐f PBPK model of ACS in the pregnant population

**FIGURE 3:**
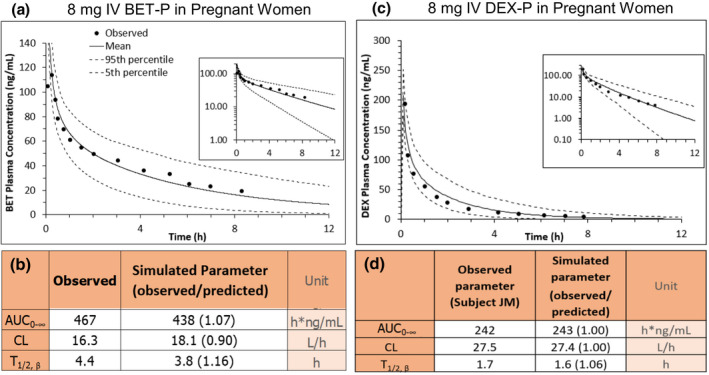
Verification of our m‐f PBPK model in the pregnant White population after i.v. administration of ACS as evidenced by the model predictions falling within our a priori defined acceptance criteria. Predicted (mean ‐ solid lines; 5th and 95th percentile – dashed lines) and observed (circles) data after i.v. administration of (a) 8 mg of BET‐P at GW 37 (one representative subject in Petersen et al., 1983) and (c) 8 mg of DEX‐P at GW 38 (mean of 8 subjects in Tsuei et al., 1980). Insets in panels a and c show the ACS concentrations plotted on a log scale. (b, d) Comparison of observed and predicted pharmacokinetic parameters from data in a and b, respectively, show that the predicted values met our a priori defined acceptance criteria. ACS, antenatal corticosteroids; AUC, area under the concentration‐time curve; BET, betamethasone; BET‐P, betamethasone phosphate; CL, clearance; DEX, dexamethasone; DEX‐P, dexamethasone phosphate; GW, gestational weight; m‐f PBPK, maternal‐fetal physiologically‐based pharmacokinetic; T_1/2_, terminal half‐life

**FIGURE 4:**
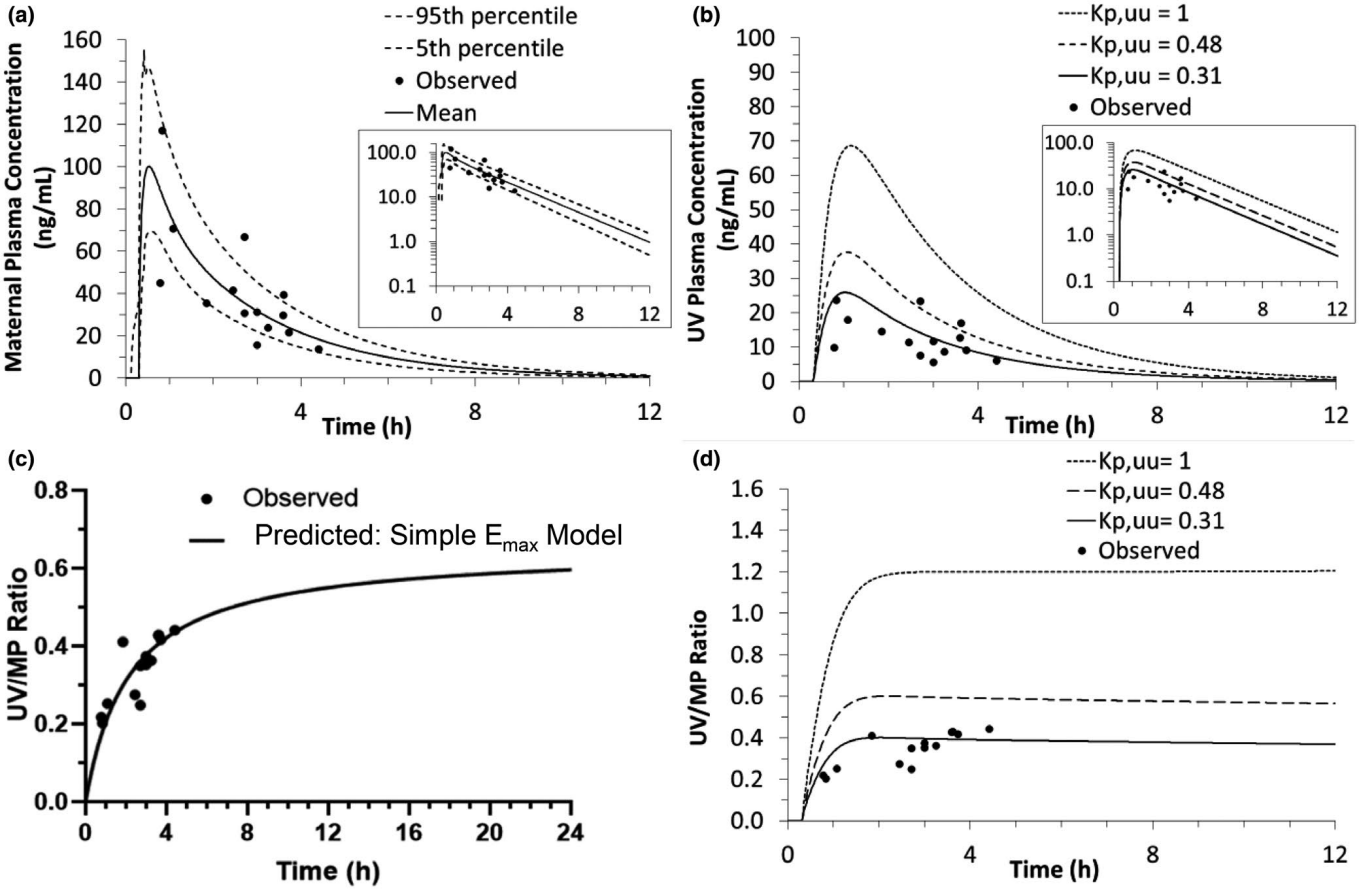
The m‐f PBPK model predictions (after i.m. DEX‐P administration) of DEX maternal and UV plasma concentration‐time profiles as well as UV/MP ratio with and without placental CL_int,Pgp,placenta_ incorporated into our m‐f PBPK model. (a) The m‐f PBPK model predicted mean maternal plasma concentration‐time profile (solid line) well described the observed maternal concentrations at the time of delivery (circles; pooled from 14 mothers at GW38) after optimization of *k_a_* (3 h^−1^) and *t*_lag_ (0.3 h) (AAFE = 1.3). (b) The m‐f PBPK model predicted UV plasma concentration‐time profile with CL_int,Pgp,placenta_ (*K_p,uu_* = 0.48, dashed line and *K_p,uu_* = 0.31, solid line) better described the observed UV plasma concentration‐time profile (black circles) than when placental P‐gp was not incorporated into the model (*K_p,uu_* = 1, dotted line) (UV: AAFE*_Kp,uu_* _= 1_ = 3.46; AAFE*_Kp,uu_* _= 0.48_ = 1.8, AAFE*_Kp,uu_* _= 0.31_ = 1.43). In panels a and b, insets show DEX plasma concentrations plotted on a log scale. (c) Predicted plateau value (0.59) of the observed UV/MP ratio determined by fitting the simple E_max_ model (black line) to the observed data (black circles). This plateau value translates to a *K_p,uu_* = 0.48 (d) the m‐f PBPK model predicted UV/MP ratios without placental CL_int,Pgp,placenta_ (*K_p,uu_* = 1, dotted line), with CL_int,Pgp,placenta_ derived from panel c (*K_p,uu_* = 0.48, dashed line) or one that allows the model to best describe the observed UV/MP ratios (*K_p,uu_* = 0.31, solid line; UV/MP: AAFE*_Kp,uu_* _= 1_ = 3.30; AAFE*_Kp,uu_* _= 0.48_ = 1.64, AAFE*_Kp,uu_* _= 0.31_ = 1.17). Observed data from Tsuei et al., 1980 are shown as filled circles (8 mg DEX‐P i.m. at GW38). Simulated mean profiles are shown as solid lines, 5th and 95th percentile profiles are shown as dotted lines. For the fetus, see Figure S1 for the 5th and 95th percentile profiles. AAFE, absolute average fold error; DEX, dexamethasone; DEX‐P, dexamethasone phosphate; E_max_, maximum effect; GW, gestational weight; m‐f PBPK, maternal‐fetal physiologically‐based pharmacokinetic; MP, maternal vein; UV, umbilical vein

**FIGURE 5:**
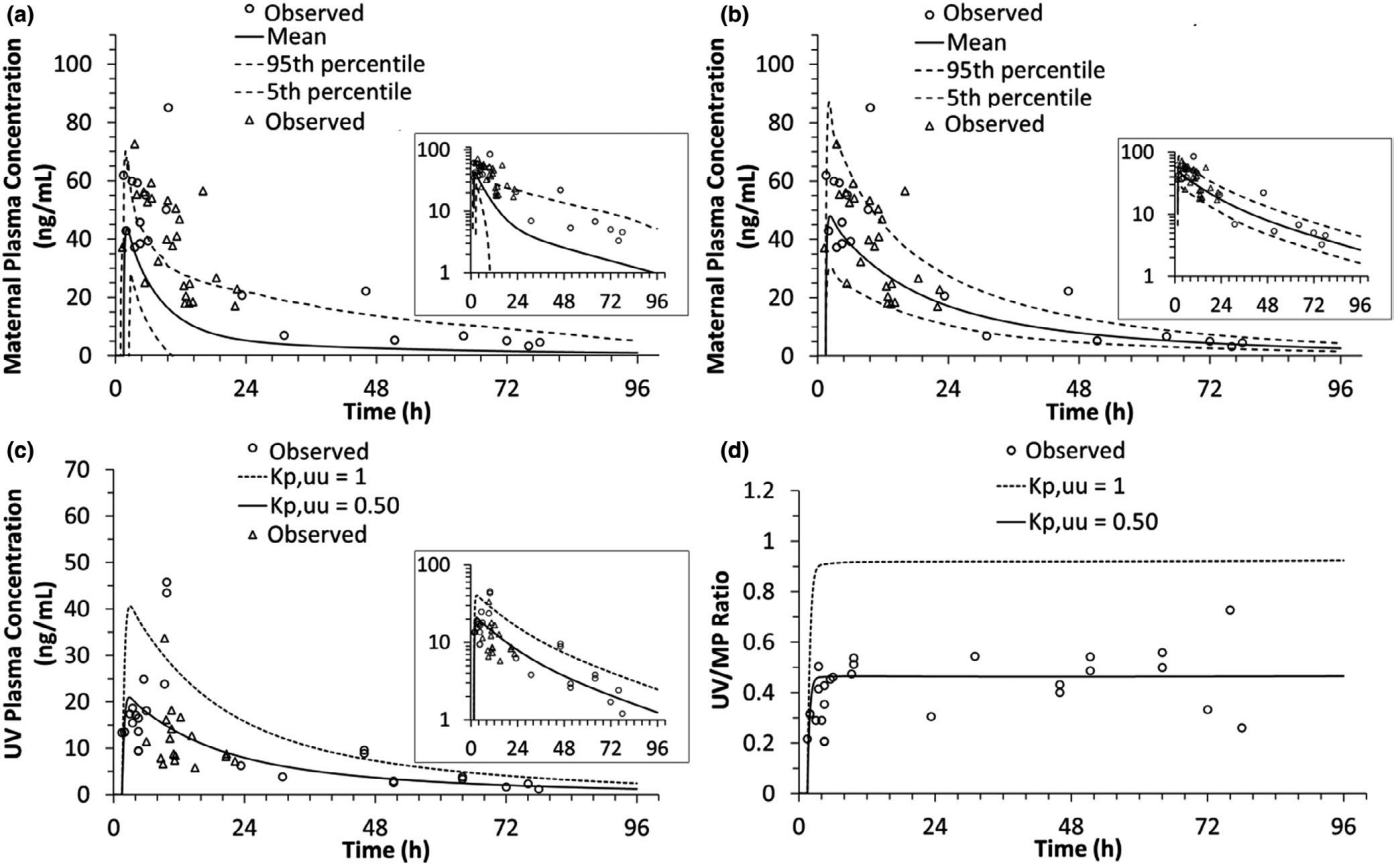
The m‐f PBPK model predictions (after i.m. BET‐P:A) of BET maternal and UV plasma concentration‐time profiles as well as UV/MP ratio with and without CL_int,Pgp,placenta_ incorporated into our m‐f PBPK model. (a) Predicted mean maternal plasma concentration‐time profile (solid line) with CL_hep,int_ (62 L/h); (a 2‐fold increase of clearance compared to non‐pregnant individuals). Maternal simulated plasma concentration‐time profile poorly predicted the observed data (AAFE = 2.03). The observed data from Ballabh et al., 2002 and Foissac et al., 2020 were pooled from 56 mothers at the time of delivery (12 mg BET‐P:A i.m. at GW 32). The open triangles and circles were from 25 and 31 women, respectively. The 5th and 95th percentile profiles are shown as dotted lines. (b) When CL_hep,int_ was reduced to 22 L/h, the predicted mean maternal plasma concentration‐time profile described the observed data better (AAFE = 1.41). The predicted maternal plasma concentration‐time profiles incorporated dual rates of absorption *k_a_* (*k_a_*_1_ = 1.5 and *k_a_*_1_ = 0.2 h^−1^) and a *t*_lag_ (1.5 h). (c) Predicted mean umbilical vein C ‐T profile with CL_int,Pgp,placenta_ incorporated into the model (*K_p,uu_* = 0.50, solid line) better described the observed concentrations than predictions without CL_int,Pgp,placenta_ (*K_p,uu_* = 1, dotted line; AAFE*_Kp,uu_* _= 1_ = 2.19; AAFE*_Kp,uu_* _= 0.5_ = 1.47). For fetal 5th and 95th percentile profiles see Figure S1. (d) Predicted UV/MP ratios with (*K_p,uu_* = 0.50; AAFE*_Kp,uu_* _= 0.50_ =1.26) and without CL_int, Pgp,placenta_ (*K_p,uu_* = 1; AAFE*_Kp,uu_* _= 1_ = 2.22) demonstrate that CL_int,Pgp,placenta_ is necessary to explain the observed data. AAFE, absolute average fold error; BET, betamethasone; BET‐P:A, betamethasone phosphate:acetate; CL, clearance; GW, gestational weight; m‐f PBPK, maternal‐fetal physiologically‐based pharmacokinetic; MP, maternal vein; UV, umbilical vein

Consistent with our previous observations,^20^ to predict the i.v. plasma concentration‐time profiles of the ACS in the White population at term, we assumed that CYP3A‐mediated hepatic intrinsic CL of the ACS was induced twofold by pregnancy (i.e., DEX CL_hep,int_ = 106 L/h, BET CL_hep,int_ = 62 L/h). Using our acceptance criteria, these model‐predicted plasma concentration‐time profiles were successfully verified by comparing them with the observed profiles after i.v. administration of BET‐P (Figure 3a,b) or DEX‐P (Figure 3c,d). Then, the m‐f PBPK model‐predicted plasma concentration‐time profiles of the ACS were compared with the observed data (Tsuei et al. 1980) after i.m. administration of DEX‐P to pregnant women at term (one data point per subject; Figure 4a). For DEX, the values for *k_a_* (2.85 h^−1^) and *T*_lag_ (0.2 h) were optimized to better describe the observed data (absolute average fold error [AAFE] = 1.3). In contrast, simulating a twofold induction of BET CL_hep,int_ failed to predict maternal concentrations after i.m. administration of the BET‐P:A mixture (AAFE = 2.03; one data point per subject; Figure 5a). Surprisingly, the CL_hep,int_ that accurately described maternal BET concentrations with an AAFE of 1.41 was 11.2 L/h, a value much lower than that in the non‐pregnant population (Figure 5b).

*Optimization of DEX and BET K_p,uu_ through sensitivity analysis*

For BET, in vivo placental efflux clearance yielding *K_p,uu_* = 0.5 resulted in the best match between the predicted and observed UV/MP ratio (AAFE*_Kp,uu_* _= 0.5_ = 1.47) versus when no CL_Pgp,placenta_ was invoked (AAFE*_Kp,uu_* _= 1_ = 2.19) (Figure 5d). For DEX, the theoretical UV/MP ratio plateau was estimated as 0.59 using the simple maximum effect (E_max_) model (Figure 4c). This value improved model predictions of UV/MP ratios compared to when CL_Pgp,placenta_ of the drug was not incorporated in the model (AAFE*_Kp,uu_* _= 0.48_ = 1.8; AAFE*_Kp,uu_* _= 1_ = 3.46; Figure 4d). Furthermore, adjusting the value of *K_p,uu_* to 0.31 (90% confidence interval [CI] = 0.20–0.42) allowed the model predicted UV/MP ratios to best match the observed values (AAFE*_Kp,uu_* _= 0.31_ = 1.43). To develop alternative dosing regimens of the ACS (described below), BET *K_p,uu_* = 0.5 (90% CI = 0.29–0.71) and DEX *K_p,uu_* = 0.48 (90% CI = 0.30–0.66) were used. The use of the latter is justified in the Discussion section.

### Designing alternative ACS dosing regimens (i.m.) at GW30 using our m‐f PBPK model

**FIGURE 6:**
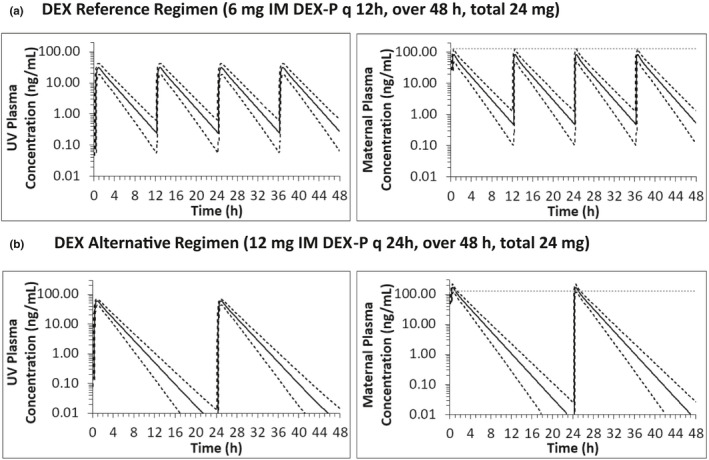
Predicted fetal and maternal UV plasma concentration‐time profiles at GW 30 for i.m. DEX‐P reference (a) and an alternative (b) dosing regimen using our final m‐f PBPK model. (a) The reference dosing regimen resulted in fetal AUC_0–48_ of 342 ng*h/ml, UV 5th percentile *C*_min_ of 0.06 ng/ml (dashed line) and mean UV *C*_min_ of 0.24 ng/ml (solid line). The corresponding maternal values were 771 ng*h/ml for AUC_0–48_ and 129.6 ng/ml 95t^h^ percentile C_max_ (dashed line). (b) The alternative dosing regimen maintained fetal AUC_0–48_ of 342 ng/ml, decreased fetal UV 5th percentile *C*_min_ to less than 0.01 ng/ml. The maternal 95th percentile C_max_ (259.0 ng/ml) increased twofold while maintaining AUC_0–48_ at 771 ng*h/ml. Predicted mean plasma concentration‐time profiles are solid lines, 5th and 95th percentiles are dashed lines. Horizontal dotted lines in maternal plasma concentration‐time profiles denote maximum targeted cutoff value for maternal 95th percentile C_max_ (129.6 ng/ml defined by the reference dosing regimen). These *C*_min_ and C_max_ values are absolute values determined over the entire 48 h period. AUC, area under the concentration‐time curve; C_max_, maximum plasma concentration; C_min_, minimum plasma concentration; DEX, dexamethasone; DEX‐P, dexamethasone phosphate; GW, gestational weight; m‐f PBPK, maternal‐fetal physiologically‐based pharmacokinetic; UV, umbilical vein

For the alternative ACS dosing regimens, maternal and fetal C_max_, minimum plasma concentration (*C*_min_), and AUC_0–48_ over the entire 48 h dosing regimen were computed. DEX‐P alternative regimen (Figure 6b) of 12 mg administered every 24 h (vs. the reference regimen of 6 mg i.m. q 12 h; Figure 6a) maintained fetal AUC_0–48_ of the reference regimen (342 ng*h/ml). Fetal fifth percentile *C*_min_ decreased from 0.06 to less than 0.01 ng/ml. This alternative regimen also resulted in a twofold increase in maternal 95th percentile C_max_ (259.0 ng/ml) compared to the 95th percentile C_max_ (129.6 ng/ml) for the reference regimen. As expected, the total maternal exposure (AUC_0–48_) remained at 771 ng*h/ml equal to that for the reference regimen.

**FIGURE 7:**
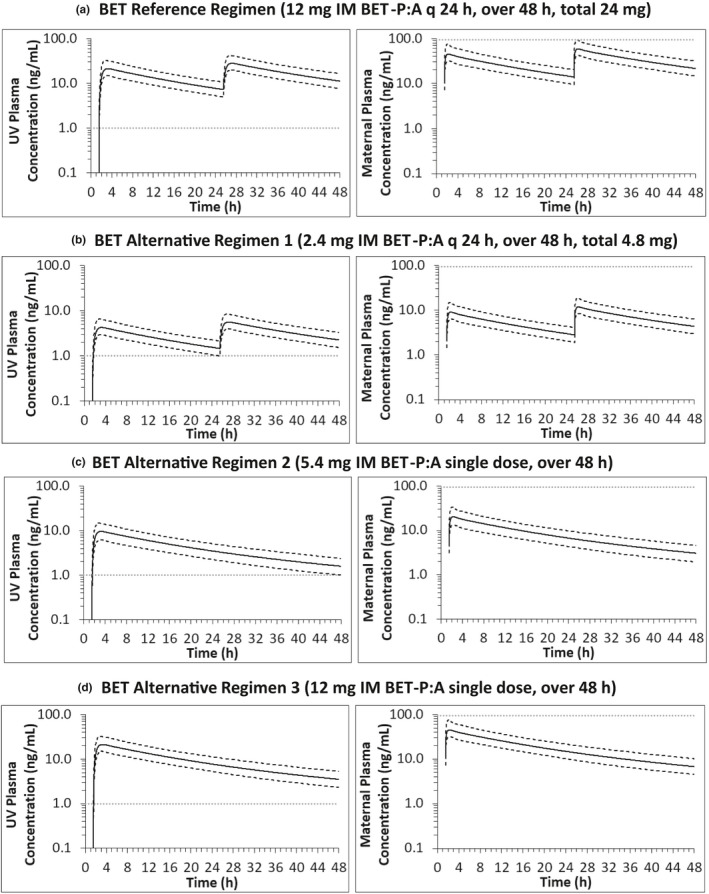
Predicted fetal and maternal UV plasma concentration‐time profiles at GW 30 for the IM BET‐P:A reference (a) and an alternative (b–d) dosing regimens using our final m‐f PBPK model (a) The reference dosing regimen resulted in greater than 1 ng/ml fetal UV 5th percentile *C*_min_ (5 ng/ml) and mean UV *C*_min_ (7.3 ng/ml). Fetal AUC_0–48_ was 724 ng*h/ml. The corresponding maternal values were 94 ng/ml and 1424 ng*h/ml, respectively. (b) The alternative dosing regimen 1 decreased fetal UV 5th percentile *C*_min_ by from 5 to 1 ng/ml and maintained UV plasma concentration above 1 ng/ml for the duration of drug administration (48 h). Fetal AUC_0–48_ decreased to 145 ng*h/ml, maternal 95th percentile C_max_ to 18.7 ng/ml and maternal AUC_0–48_ to 285 ng*h/ml. All listed parameters as well as the total dose decreased by 80% compared to reference dosing regimen. (c) The alternative dosing regimen two decreased fetal UV 5th percentile *C*_min_ by 80% (from 5 to 1 ng/ml) and maintained UV plasma concentration above 1 ng/ml for the duration of drug administration (48 h). Fetal AUC_0–48_ decreased 74% to 191 ng*h/ml. The maternal 95th percentile C_max_ (33.7 ng/ml) decreased 64% and the AUC_0–48_ (375 ng*h/ml) decreased 74%. (d) The alternative dosing regimen three (currently used in BETADOSE clinical trial, see Schmitz et al., 2019) decreased fetal UV 5th percentile *C*_min_ by 53% (from 5 to 2.3 ng/ml) and maintained UV plasma concentration above 1 ng/ml for the duration of drug administration (48 h). Fetal AUC_0–48_ decreased 41% to 424 ng*h/ml. The maternal 95th percentile C_max_ (75 ng/ml) decreased 22% and the AUC_0–48_ (833 ng*h/ml) decreased 42%. Predicted mean plasma concentration‐time profiles are solid lines, 5th and 95th percentiles are dashed lines. Horizontal dotted line in maternal plasma concentration‐time profiles denote maximum cutoff value for maternal 95th percentile C_max_ (94 ng/ml defined by the reference dosing regimen). Horizontal dotted line in fetal plasma concentration‐time profiles denote minimum cut‐off value for fetal 5th percentile *C*_min_ (1 ng/ml). These *C*_min_ and C_max_ values are absolute values determined over the entire 48 h period. AUC, area under the concentration‐time curve; BET, BET, betamethasone; BET‐P:A, betamethasone phosphate:acetate;; BET‐P:A, BET phosphate:acetate; C_max_, maximum plasma concentration; C_min_, minimum plasma concentration; GW, gestational weight; m‐f PBPK, maternal‐fetal physiologically‐based pharmacokinetic; UV, umbilical vein

Reducing the reference BET‐P:A dose to 2.4 mg q 24 h for 48 h (regimen 1; administered as often as the reference regimen; 20% of the total reference dose; Figure 7a) showed an 80% decrease in fetal AUC_0–48_ (145 ng*h/ml) and maintained fetal drug plasma concentrations greater than 1 ng/ml for 48 h. Fetal fifth percentile *C*_min_ of 1 ng/ml was observed at the 48 h time point and decreased by 80%, compared to reference regimen (Figure 7b). Maternal 95th percentile C_max_ (18.7 ng/ml) and maternal AUC_0–48_ (285 ng*h/ml) also decreased by 80% in comparison to the BET reference regimen values of 94 ng/ml and 1424 ng*h/ml (Figure 7b).

BET‐P:A alternative dosing regimen two (Figure 7c) of single 5.4 mg dose (22.5% of the total reference dose; Figure 7a) decreased fetal AUC_0–48_ by 74% from 724 to 191 ng*h/ml. Fetal 5th percentile *C*_min_ decreased by 80% from 5 to 1 ng/ml and remained greater than 1 ng/ml for 48 h. Maternal 95th percentile C_max_ (33.7 ng/ml) decreased 64% versus the BET reference regimen (94 ng/ml). Total maternal mean AUC decreased 74% (375 ng*h/ml).

We also predicted BET plasma concentrations for a dosing regimen currently used in BETADOSE clinical trial.^21^ BET‐P:A alternative dosing regimen three (Figure 7d) of single 12 mg dose, 50% of the total reference dose (Figure 7a), decreased fetal AUC_0–48_ by 41% from 724 to 424 ng*h/ml and fetal fifth percentile *C*_min_ by 53% from 5 to 2.3 ng/ml but remained greater than 1 ng/ml for 48 h. Maternal 95th percentile C_max_ (75 ng/ml) decreased by 22% from the BET reference regimen (94 ng/ml). Total maternal AUC_0–48_ decreased 42% (833 ng*h/ml).

## DISCUSSION

Respiratory failure due to RDS is one of the most common causes of death in premature infants in US neonatal intensive care units. Even among infants who survive, RDS is associated with a twofold increased risk of cerebral palsy and a 1.4‐fold increased risk of epilepsy.^22^, ^23^ Safe and effective ACS dosing is critical to reduce morbidity and mortality due to preterm delivery. In the present work, for the first time, we used our m‐f PBPK model to predict and then verify maternal‐fetal exposure to ACS. Then, to illustrate the utility of m‐f PBPK model, we used it to propose alternative ACS dosing regimens that could potentially maximize ACS efficacy while minimizing ACS toxicity or could be more convenient to implement in the clinic due to reduced frequency of administration.

To develop our ACS m‐f PBPK model, we first ensured that our PBPK model could describe ACS exposure after i.m. administration to non‐pregnant Indian population (Figure 2). It could, as evidenced by successful model verification of the observed data (Figure 2c,d). However, our model modestly overpredicted BET CL in this population perhaps due to ethnic differences in BET CL. This was not of concern to us as the sole reason for use of this data set was to allow us to estimate the dual *k_a_* for BET‐P:A necessary to describe two distinct absorption (or release) phases of the phosphate and acetate prodrug. Once the PBPK model was verified for the non‐pregnant population, the drug‐dependent parameters were fixed and our m‐f PBPK model was populated with these parameters. Then, we verified our m‐f PBPK model after i.v. administration of ACS in pregnancy (Figure 3). To do so, we incorporated our previously reported twofold induction of hepatic CYP3A4 activity at term.^20^ BET and DEX are cleared from the body predominately by CYP3A metabolism.^24^, ^25^ Indeed, this magnitude of induction was consistent with the observed twofold increase in midazolam CL (a selective CYP3A probe) during the third trimester (Figure 3). Although others have reported different magnitudes of CYP3A induction in the third trimester, these findings have been based on studies where a selective CYP3A probe was not utilized.^26^, ^27^, ^28^ Indeed, i.v. BET‐P C‐T profiles in pregnant women were well explained by this twofold induction of hepatic CYP3A activity (Figure 3a). Therefore, surprisingly, the observed clearance after i.m. BET‐P:A administration in pregnancy (5.7 L/h^13^) was lower than after i.v. BET‐P administration to the non‐pregnant population (16.3 L/h; Figure 5a). The reasons for this decrease (not increase) are not clear and should be explored further. Because the goal of this study was to predict fetal rather than maternal drug plasma concentrations, it was important to accurately describe maternal BET plasma concentration‐time profiles. Hence, we decreased BET maternal CL_hep,int_ to best describe BET maternal plasma concentration‐time profile after intramuscular BET‐P:A administration (Figure 5b).

In order to accurately predict fetal DEX/BET plasma concentration‐time profiles, it is important to account for the processes that govern transplacental transfer of these drugs into the fetus (i.e., passive diffusion and active placental efflux clearances). Because both drugs are substrates of P‐gp,^10^, ^11^ which is highly abundant in placenta,^15^, ^29^, ^30^, ^31^ we aimed to optimize their magnitude of P‐gp mediated efflux to obtain fetal drug exposure (in vivo *K_p,uu_*) that agrees with the observed data. Our PBPK modeling showed that the in vivo CL_int,PD,placenta_ for both drugs was indeed large, and limited by placental blood flow (~45 L/h in third trimester). Moreover, not accounting for placental efflux resulted in significant overestimation of fetal drug exposure and therefore underestimation of the dose needed to administer to the mother to achieve fetal plasma concentration of greater than 1 ng/ml.

The obtained *K_p,uu_* value for BET (0.5) was determined with greater confidence (Figure 5) than for DEX (*K_p,uu_* = 0.48 or 0.31; Figure 4) due to excellent agreement of our model predicted BET UV/MP ratio with that observed.^13^, ^32^, ^33^ In preliminary Transwell efflux studies of DEX and BET in P‐gp and BCRP overexpressing MDCKII cells, we found similar in vitro P‐gp mediated efflux ratios for the two ACS and no BCRP‐mediated transport, respectively (unpublished data). These data strongly suggests that the in vivo *K_p,uu_* of these drugs should be similar. This is not surprising as these two ACS are epimers and P‐gp does not readily discriminate between isomers. For this reason, to develop alternative dosing regimens for DEX, we used *K_p,uu_* = 0.48, a value close to that of BET. However, the in vivo DEX UV/MP data suggest that DEX *K_p,uu_* could just as well be 0.31. To resolve this discrepancy, additional in vivo DEX data sets are needed to better define its in vivo *K_p,uu_*.

The efficacy of the ACS reference regimens in reducing RDS is modest. When the reference ACS regimens are used, the relative risk of RDS (compared with placebo) ranges from 0.6 to 1.16^34^, ^35^, ^36^, ^37^ suggesting a need to optimize the ACS dosing regimens. However, increasing the ACS dosing rate could potentially enhance ACS toxicity. For example, when the reference DEX‐P regimen is used, maternal infections significantly increase from 6% in the placebo arm to 10% in the ACS arm (odds ratio of 1.64).^38^ Likewise, there are concerns of long‐term neonatal neurodevelopmental toxicity from use of the ACS.^6^, ^7^, ^39^ Therefore, to demonstrate the in vivo clinical application of our m‐f PBPK model, we used it to devise alternative dosing regimens for ACS that could enhance their efficacy while minimizing their toxicity. It is not clear whether the efficacy and toxicity of these ACS is related to their maternal‐fetal exposure (AUC) or C_max_ or both. In the absence of this information when designing alternative ACS dosing regimens, we took the conservative approach of not exceeding the reference regimen ACS maternal‐fetal exposure (AUC) or C_max_ or both. In addition, for BET only, we designed dosing regimens based on maintaining fetal plasma concentration greater than 1 ng/ml based on the efficacy data in sheep.^18^ When designing these alternative dosing regimens, we also took into consideration the dosing frequency of the regimen so that it was convenient to implement in the clinic (not more frequent than twice a day).

To design a convenient alternative dosing regimen for i.m. DEX‐P (Figure 6b), fulfilling criterion three in the Method section, we reduced the number of doses because the reference regimen is already administered q 12 h (Figure 6a). Administering a twofold higher dose, but less frequently (q 24 h) helped us maintain maternal and fetal AUCs (fulfilled criterion 1a and 2). Consequently, maternal peak concentrations rose, which in the clinic may increase efficacy but produce higher incidence of adverse events than observed after reference dosing regimen (maternal infection rate of 5%–6%^34^). Due to the lack of animal or human data on fetal efficacy after lower i.m. DEX‐P doses, we refrained from designing a regimen that would decrease fetal (and maternal) AUC. However, when such data are available, our model could be used to design such dosing regimens.

To design a convenient alternative dosing regimen for IM BET‐P:A (criterion 3; Figure 7b), we could increase (q 12 h), maintain (q 24 h; Figure 7b) or decrease (single dose over 48 h; Figure 7c,d) the number of administered doses. Increasing the number of doses (to q 12 h) to maintain maternal and fetal AUC, as for DEX‐P above, produced similar maternal and fetal C_max_ and *C*_min_ as in reference regimen, and was therefore not considered further. Availability of sheep data^18^, ^40^ gave us a new guideline for efficacious fetal drug plasma concentrations greater than 1 ng/ml (criterion 2b). These data allowed us to decrease BET‐P:A dose from 12 to 2.4 mg q 24 h (Figure 7b) or to 5.4 mg single dose (Figure 7c) and fulfill criteria 1b and 2. These decreased doses resulted in decreases in fetal AUC and C_max_ and relied on the assumption that human efficacious BET plasma concentrations are equal to that in sheep. This assumption and proposed dosing regimens need to be assessed in the clinic to ensure fetal therapeutic benefit. The ongoing BETADOSE clinical trial^21^ is exploring the efficacy and safety of another BET‐P:A dosing regimen that reduces total administered i.m. dose (12 mg i.m. BET‐P:A administered as a single dose). Simulations of maternal‐fetal drug plasma concentrations for this dosing regimen are provided as alternative dosing regimen three (Figure 7d). Studies to verify these predictions are urgently needed to promote the optimal dose of ACS to administer for preterm labor, especially in low‐to‐middle income countries, where rates of preterm birth are high. Overall, lower BET drug plasma concentrations, smaller fluctuations, and less frequent administration make it more attractive therapeutic option than DEX, but this conclusion should be further evaluated in the clinic.

There are several limitations to this study, most of which are related to the limited clinical data in maternal‐fetal pairs available for these ACS. First, maternal‐fetal PK data on these ACS are limited (especially for DEX‐P) and therefore any inaccuracies in the published data will result in inaccuracies in the predicted dosing regimens. Second, limited clinical data (including PKs) are available for different ethnic populations, especially those from countries where preterm delivery rates and thus ACS use are high. DEX and BET CL for the White non‐pregnant population was 15 L/h and 7–17 L/h,^19^ higher than the value for the Indian non‐pregnant women (DEX: 9–10 L/h; BET: 5–6 L/h; Figure 2b,d,f^41^). A similar observation has been made for nifedipine, another CYP3A substrate.^42^ Therefore, we used PK parameters from the White non‐pregnant population and verified our m‐f PBPK model with data from White pregnant women. Hence, PK/PD studies in pregnant Indian women are needed and are underway.^34^ One such study, conducted by the World Health Organization, is investigating the efficacy of i.m. BET‐P at 2 mg q 12 h for 48 h. Therefore, for comparison, we predicted the maternal‐fetal exposure to BET for this dosing regimen (Figure S2). If this regimen is found to be efficacious as the BET‐P:A reference regimen, it has the potential to reduce maternal‐fetal risks. Ideally, future studies will produce high‐quality data sets with maternal‐fetal paired sampling, that include accurate recording of time post last dose, and stabilization of hydrolysis of the ACS prodrugs after collection. We should note here that the in vivo cleavage of BET‐P to the BET is rapid and appears to be complete within 60 min.^43^ Corresponding data for DEX‐P or BET‐A are not available. Because in vivo hydrolysis of BET‐A is slower than BET‐P, if a substantial amount of BET‐A is present in the drawn blood sample and is subsequently hydrolyzed to BET prior to freezing the plasma, this could potentially explain the lower than anticipated clearance of BET in pregnant women when BET‐P:A is administered but not when BET‐P is administered. Parenthetically, the ACS UV/MP data used here were obtained at least 60 min after ACS prodrug administration.

To our knowledge, this is the first study to estimate *K_p,uu_*, CL_int, Pgp,placenta_, and CL_int,PD, placenta_ of a placental P‐gp effluxed drug from the observed UV/MP data and then to dynamically predict fetal plasma concentration of the drug, in this case the ACS. Then, these values were populated in our m‐f PBPK model to simulate maternal‐fetal ACS exposure, for various ACS dosing regimens, during pregnancy. Our approach and our m‐f PBPK model could be used in the future to predict maternal‐fetal exposure to any drug and to devise alternative dosing regimens (including those not described here) of the drug to guide drug therapy of the maternal‐fetal dyad.

## CONFLICT OF INTEREST

M.M. is an owner of Milad Pharmaceutical Consulting LLC, and a paid consultant/employee of Certara USA Inc. (Princeton, NJ) and the Bill & Melinda Gates Foundation. All other authors declared no competing interests for this work.

## AUTHOR CONTRIBUTIONS

O.A., M.A.M., and J.D.U. wrote the manuscript. O.A., M.A.M., and J.D.U. designed the research. O.A. performed the research. O.A. analyzed the data.

## Supporting information

Supinfo1

Supinfo2

Supinfo3

Supinfo4

Supinfo5

Supinfo6

## References

[1] Scaffidi J, Mol BW, Keelan JA. The pregnant women as a drug orphan: a global survey of registered clinical trials of pharmacological interventions in pregnancy. BJOG. 2017;124(1):132‐140. 27297096 10.1111/1471-0528.14151

[2] Zhang Z, Imperial MZ, Patilea‐Vrana GI, Wedagedera J, Gaohua L, Unadkat JD. Development of a novel maternal‐fetal physiologically based pharmacokinetic model I: insights into factors that determine fetal drug exposure through simulations and sensitivity analyses. Drug Metab Dispos. 2017;45(8):920‐938. 28588050 10.1124/dmd.117.075192 PMC5506457

[3] Zhang Z, Unadkat JD. Development of a novel maternal‐fetal physiologically based pharmacokinetic model II: verification of the model for passive placental permeability drugs. Drug Metab Dispos. 2017;45(8):939‐946. 28049636 10.1124/dmd.116.073957 PMC5506455

[4] Vogel JP, Oladapo OT, Pileggi‐Castro C, et al. Antenatal corticosteroids for women at risk of imminent preterm birth in low‐resource countries: the case for equipoise and the need for efficacy trials. BMJ Global Health. 2017;2(3):e000398. 10.1136/bmjgh-2017-000398 PMC5656119 29082019

[5] Roberts D, Brown J, Medley N, Dalziel SR. Antenatal corticosteroids for accelerating fetal lung maturation for women at risk of preterm birth. Cochrane Database Syst Rev. 2017;3:CD004454. 28321847 10.1002/14651858.CD004454.pub3 PMC6464568

[6] Räikkönen K, Gissler M, Kajantie E. Maternal antenatal corticosteroid treatment and childhood mental and behavioral disorders—reply. JAMA. 2020;324(15):1570. 10.1001/jama.2020.15449 33079149

[7] Crowther CA, Doyle LW, Haslam RR, et al. Outcomes at 2 years of age after repeat doses of antenatal corticosteroids. N Engl J Med. 2007;357(12):1179‐1189. 17881750 10.1056/NEJMoa071152

[8] Boland EW. Clinical comparison of the newer anti‐inflammatory corticosteroids. Ann Rheum Dis. 1962;21(2):176‐187. 13870748 10.1136/ard.21.2.176 PMC1007268

[9] American College of O, Gynecologists’ Committee on Practice B‐O . Practice Bulletin No. 171: management of preterm labor. Obstet Gynecol. 2016;128(4):e155‐e164. 27661654 10.1097/AOG.0000000000001711

[10] Crowe A, Tan AM. Crowe A, Tan AM. Oral and inhaled corticosteroids: differences in P‐glycoprotein (ABCB1) mediated efflux. Toxicol Appl Pharmacol. 2012;260(3):294‐302. 22464980 10.1016/j.taap.2012.03.008

[11] Yates CR, Chang C, Kearbey JD, et al. Structural determinants of P‐glycoprotein‐mediated transport of glucocorticoids. Pharm Res. 2003;20(11):1794‐1803. 14661924 10.1023/b:pham.0000003377.39548.f6

[12] Tsuei SE, Petersen MC, Ashley JJ, McBride WG, Moore RG. Disporition of synthetic glucocorticoids. II. Dexamethasone in parturient women. Clin Pharmacol Ther. 1980;28(1):88‐98. 7389259 10.1038/clpt.1980.136

[13] Ballabh P, Lo ES, Kumari J, et al. Pharmacokinetics of betamethasone in twin and singleton pregnancy. Clin Pharmacol Ther. 2002;71(1):39‐45. 11823756 10.1067/mcp.2002.120250

[14] Foissac F, Zheng Y, Hirt D, et al. Maternal betamethasone for prevention of respiratory distress syndrome in neonates: population pharmacokinetic and pharmacodynamic approach. Clin Pharmacol Ther. 2020;108(5):1026‐1035. 32394434 10.1002/cpt.1887

[15] Anoshchenko O, Prasad B, Neradugomma NK, Wang J, Mao Q, Unadkat JD. Gestational age–dependent abundance of human placental transporters as determined by quantitative targeted proteomics. Drug Metab Dispos. 2020;48(9):735‐741. 32591415 10.1124/dmd.120.000067 PMC7469251

[16] Tsuei SE, Moore RG, Ashley JJ, McBride WG. Disposition of synethetic glucocorticoids. I. Pharmacokinetics of dexamethasone in healthy adults. J Pharmacokinet Biopharm. 1979;7(3):249‐264. 480147 10.1007/BF01060016

[17] Petersen MC, Nation RL, McBride WG, Ashley JJ, Moore RG. Pharmacokinetics of betamethasone in healthy adults after intravenous administration. Eur J Clin Pharmacol. 1983;25(5):643‐650. 6662164 10.1007/BF00542353

[18] Schmidt AF, Kemp MW, Rittenschober‐Bohm J, et al. Schmidt AF, Kemp MW, Rittenschober‐Böhm J, Kannan PS, Usuda H, Saito M, Furfaro L, Watanabe S, Stock S, Kramer BW, Newnham JP, Kallapur SG, Jobe AH. Low‐dose betamethasone‐acetate for fetal lung maturation in preterm sheep. Am J Obstet Gynecol. 2018;218(1):132.e1‐132.e9. 29138038 10.1016/j.ajog.2017.11.560 PMC5759749

[19] Ke AB, Milad MA. Evaluation of maternal drug exposure following the administration of antenatal corticosteroids during late pregnancy using physiologically‐based pharmacokinetic modeling. Clin Pharmacol Ther. 2019;106(1):164‐173. 30924921 10.1002/cpt.1438

[20] Zhang Z, Farooq M, Prasad B, Grepper S, Unadkat JD. Prediction of gestational age‐dependent induction of in vivo hepatic CYP3A activity based on HepaRG cells and human hepatocytes. Drug Metab Dispos Biol Fate Chem. 2015;43(6):836‐842. 25802327 10.1124/dmd.114.062984 PMC4429679

[21] Schmitz T, Alberti C, Ursino M, et al. Full versus half dose of antenatal betamethasone to prevent severe neonatal respiratory distress syndrome associated with preterm birth: study protocol for a randomised, multicenter, double blind, placebo‐controlled, non‐inferiority trial (BETADOSE). BMC Pregnancy Childbirth. 2019;19(1):67. 30755164 10.1186/s12884-019-2206-x PMC6373166

[22] Dyer J. Neonatal respiratory distress syndrome: tackling a worldwide problem. P T. 2019;44(1):12‐14. 30675087 PMC6336202

[23] Thygesen SK, Olsen M, Pedersen L, Henderson VW, Ostergaard JR, Sorensen HT. Respiratory distress syndrome in preterm infants and risk of epilepsy in a Danish cohort. Eur J Epidemiol. 2018;33(3):313‐321. 28887607 10.1007/s10654-017-0308-1

[24] Varis T, Kivisto KT, Backman JT, Neuvonen PJ. The cytochrome P450 3A4 inhibitor itraconazole markedly increases the plasma concentrations of dexamethasone and enhances its adrenal‐suppressant effect. Clin Pharmacol Ther. 2000;68(5):487‐494. 11103751 10.1067/mcp.2000.110772

[25] Gentile DM, Tomlinson ES, Maggs JL, Park BK, Back DJ. Dexamethasone metabolism by human liver in vitro. Metabolite identification and inhibition of 6‐hydroxylation. J Pharmacol Exp Ther. 1996;277(1):105‐112. 8613906

[26] De Sousa MM, Lui G, Zheng Y, et al. A physiologically‐based pharmacokinetic model to predict human fetal exposure for a drug metabolized by several CYP450 pathways. Clin Pharmacokinet. 2017;56(5):537‐550. 27766562 10.1007/s40262-016-0457-5

[27] Xia B, Heimbach T, Gollen R, Nanavati C, He H. A simplified PBPK modeling approach for prediction of pharmacokinetics of four primarily renally excreted and CYP3A metabolized compounds during pregnancy. AAPS J. 2013;15(4):1012‐1024. 23835676 10.1208/s12248-013-9505-3 PMC3787241

[28] Dallmann A, Ince I, Coboeken K, Eissing T, Hempel G. A physiologically based pharmacokinetic model for pregnant women to predict the pharmacokinetics of drugs metabolized via several enzymatic pathways. Clin Pharmacokinet. 2018;57(6):749‐768. 28924743 10.1007/s40262-017-0594-5

[29] Ceckova‐Novotna M, Pavek P, Staud F. P‐glycoprotein in the placenta: expression, localization, regulation and function. Reprod Toxicol. 2006;22(3):400‐410. 16563694 10.1016/j.reprotox.2006.01.007

[30] Han LW, Gao C, Mao Q. An update on expression and function of P‐gp/ABCB1 and BCRP/ABCG2 in the placenta and fetus. Expert Opin Drug Metab Toxicol. 2018;14(8):817‐829. 30010462 10.1080/17425255.2018.1499726 PMC6290921

[31] Mathias AA, Hitti J, Unadkat JD. P‐glycoprotein and breast cancer resistance protein expression in human placentae of various gestational ages. Am J Physiol Regul Integr Comp Physiol. 2005;289(4):R963‐R969. 15961534 10.1152/ajpregu.00173.2005

[32] Gyamfi C, Mele L, Wapner RJ, et al. The effect of plurality and obesity on betamethasone concentrations in women at risk for preterm delivery. Am J Obstet Gynecol. 2010;203(3):219.e1‐219.e5. Epub 2010/06/29. 20579955 10.1016/j.ajog.2010.04.047 PMC3214971

[33] Ballard PL, Granberg P, Ballard RA. Glucocorticoid levels in maternal and cord serum after prenatal betamethasone therapy to prevent respiratory distress syndrome. J Clin Investig. 1975;56(6):1548‐1554. 1202085 10.1172/JCI108236 PMC333133

[34] Collaborators WAT, Oladapo OT, Vogel JP, et al. Antenatal dexamethasone for early preterm birth in low‐resource countries. N Engl J Med. 2020;383(26):2514‐2525. 33095526 10.1056/NEJMoa2022398 PMC7660991

[35] Collaborative . Effect of antenatal dexamethasone administration on the prevention of respiratory distress syndrome. Am J Obstet Gynecol. 1981;141(3):276‐287. 7025638

[36] Gyamfi‐Bannerman C, Thom EA. Antenatal betamethasone for women at risk for late preterm delivery. N Engl J Med. 2016;375(5):486‐477. 10.1056/NEJMc1605902 27518669

[37] Liggins GC, Howie RN. A controlled trial of antepartum glucocorticoid treatment for prevention of the respiratory distress syndrome in premature infants. Pediatrics. 1972;50(4):515‐525. 4561295

[38] Althabe F, Belizan JM, McClure EM, et al. A population‐based, multifaceted strategy to implement antenatal corticosteroid treatment versus standard care for the reduction of neonatal mortality due to preterm birth in low‐income and middle‐income countries: the ACT cluster‐randomised trial. Lancet. 2015;385(9968):629‐639. 25458726 10.1016/S0140-6736(14)61651-2 PMC4420619

[39] Wapner RJ, Sorokin Y, Thom EA, et al. Single versus weekly courses of antenatal corticosteroids: evaluation of safety and efficacy. Am J Obstet Gynecol. 2006;195(3):633‐642. 16846587 10.1016/j.ajog.2006.03.087

[40] Kemp MW, Saito M, Usuda H, et al. The efficacy of antenatal steroid therapy is dependent on the duration of low‐concentration fetal exposure: evidence from a sheep model of pregnancy. Am J Obstet Gynecol. 2018;219(3):301.e1‐301.e16. 29758177 10.1016/j.ajog.2018.05.007

[41] Jobe AH, Milad MA, Peppard T, Jusko WJ. Pharmacokinetics and pharmacodynamics of intramuscular and oral betamethasone and dexamethasone in reproductive age women in India. Clin Transl Sci. 2020;13(2):391‐399. 31808984 10.1111/cts.12724 PMC7070803

[42] Ahsan CH, Renwick AG, Waller DG, Challenor VF, George CF, Amanullah M. The influence of dose and ethnic origins on the pharmacokinetics of nifedipine. Clin Pharmacol Ther. 1993;54(3):329‐338. 8375129 10.1038/clpt.1993.155

[43] Samtani MN, Schwab M, Nathanielsz PW, Jusko WJ. Stabilization and HPLC analysis of betamethasone sodium phosphate in plasma. J Pharm Sci. 2004;93(3):726‐732. 14762910 10.1002/jps.10577

