# Supplementary material for: Estimating fetal exposure to the P‐gp substrates, corticosteroids, by PBPK modeling to inform prevention of neonatal respiratory distress syndrome
Source: CPT Pharmacometrics Syst Pharmacol. 2021 Jul 23;10(9):1057–70. doi: 10.1002/psp4.12674 (PMC8452292; doi:10.1002/psp4.12674)
Supplement: Supplementary file 1 — Supinfo1 [file PSP4-10-1057-s001.docx]

**SUPPLEMENTARY INFORMATION**

**MATERIALS AND METHODS**

Optimization of SimCYP PBPK Model of ACS in the Non-pregnant Caucasian Population (Figure 1, top panel)

Our m-f PBPK model requires, as input, drug physiological and pharmacokinetic (PK) parameters in the non-pregnant population. The majority of these ACS parameters were as described before by Ke et al., 2019 [1] while a few (described below) were optimized for the non-pregnant population in SimCYP Simulator® Version 19 (SimCYP Ltd., A Certara Company, Sheffield, UK) (**Table S1**). Our acceptance criterion was to predicted PK parameter values within 0.8 – 1.25-fold of the observed values (**Figure 1**). The values for observed data were obtained from the literature using the WebPlotDigitizer (https://automeris.io/WebPlotDigitizer/) and, if some of the PK parameters were not reported, they were obtained by analyzing the digitized mean concentration-time profiles using non-compartmental analysis in Phoenix 8.1 (linear trapezoid method was employed). Due to the limited ACS PK data available in the literature, we used the observed data in the Caucasian and Indian population to train our PBPK model after IV [2, 3] and IM administration of ACS [4], respectively. The Indian population training data set was used ONLY to estimate the differential release characteristics of BET from BET-P and BET-A in the IM BET-P:A formulation. Such data are not available in the Caucasian population.

The SimCYP simulator was populated with the ACS PK parameters as follows: The dose of the ACS drug in the administered formulation (DEX-P, BET-P or BET-P:A) was adjusted for the molecular weight of the ACS. The observed clearance (CL) for DEX (14.2 L/h) [2, 3] and BET (10.3 L/h) [2, 3] were converted to hepatic intrinsic clearance (CL_hep,int_) using the well-stirred model within SimCYP Simulator®. Based on *in vivo* itraconazole CYP inhibition data [5], dexamethasone CL_hep,int_ was assigned to CYP3A4 (f_m_=0.95) and an additional minor unidentified pathway (f_m_=0.022) [1]. Betamethasone CL_hep,int_ was assigned to CYP3A4 (f_m_=0.938) based on an unpublished report cited by Ke and Milad [1]. This report showed that a selective inhibitor for CYP3A (CYP3cide) completely inhibited the depletion of DEX and BET in human liver microsomes. Meanwhile, selective inhibitors for other CYP-enzymes (1A2; 2B6; 2C8; 2C9; 2C19 ; 2D6) had no effect on their depletion. Hence, CYP3A enzymes were deemed to be the major contributors to DEX and BET clearance *in vivo*. Renal clearance of both drugs, a minor clearance pathway, comprised 0.41 L/h (f_e_ = 0.028) for DEX [2] and 0.49 L/h (f_e_ = 0.062) for BET [6]. Volume of distribution at steady-state (V_ss_) was determined from the literature as 0.72 L/kg for DEX [2] and 1.3 L/kg for BET [3] for non-pregnant individuals. Binding of the ACS to non-albumin proteins was assumed to be negligible as shown before [7]. For IM administration of DEX-P or BET-P, the absorption-rate constant (k_a_ = 2 and 1.5 h^-1^, respectively) and lag time (T_lag_ = 1 and 1.5 h, respectively) of the drugs were optimized to describe the observed profiles [4]. After IM administration of BET-P:A mixture, to take into account the different release characteristics of the phosphate and acetate prodrug, each prodrug was assigned a different absorption rate, k_a_ (1.5 and 0.2 h^-1^, respectively), from the site of administration [4]. The remaining baseline physicochemical parameters were as described before by Ke et al., 2019 [1] (Table S1).

Verification of m-f PBPK Model of ACS in the Pregnant Population (**Figure 1, 2^nd^ panel**)

The ACS baseline pharmacokinetic parameters from non-pregnant population were incorporated, without further change except as noted below, into our previously published m-f PBPK model built in MATLAB R2020a and then adjusted for gestational age-dependent changes as described by us [8] (Figure 1). Our m-f PBPK model (Matlab Model) and the SimCYP pregnancy model were confirmed at the initial stages of this work to be equivalent in terms of predictions of maternal concentrations (given the same trial design in both models). First, using our m-f PBPK model, DEX and BET plasma concentration-time profiles were predicted in pregnant women at term (GW37-38) after IV administration of DEX-P and BET-P. Maternal hepatic CYP3A4 activity was assumed to be induced 2-fold (100%) at term [9, 10]. We chose to use 100% induction of CYP3A in pregnancy because this value was obtained using a selective CYP3A substrate midazolam. In contrast, though other studies have reported varying degrees of CYP3A induction during pregnancy (3-60%), these values were obtained using drugs that are not selective probes of CYP3A enzymes [11-13]. Then, using these IV pharmacokinetic parameters, the plasma concentration-time profiles of ACS, after IM administration of DEX-P and BET-P:A, were predicted using our m-f PBPK model. The values of k_a_ and T_lag_ were optimized to describe the observed IM DEX plasma concentration-time profiles. As was the case in non-pregnant individuals, to predict the plasma concentration-time profiles after IM BET-P:A administration, distinct rates of absorption of the phosphate and the acetate from the IM site were incorporated into our model. To generate interindividual variability in the plasma concentration-time profiles, a virtual population of 100 individuals was simulated and the mean, lower 5^th^ and the upper 95^th^ percentile profiles (90% confidence interval - CI) were generated. To do so, all of the maternal system-dependent parameters used in the model were varied as per the variability assigned within SimCYP® (~30% for each parameter). Due to the lack of data on the variability in the fetal system-dependent parameters, only variability in the maternal system-dependent parameters was included. The trial designs for model verification IM DEX-P in pregnancy were obtained from Tsuei *et al.,* 1980 [14] and for IM BET-P:A from Ballabh et al., 2002 and Foissac et al., 2020 [15, 16]. BET-P:A pregnancy pharmacokinetic data from other sources were excluded due to the lack of information on time of sampling post last dose [17, 18]. Our predictions were evaluated by computing the absolute average fold error (AAFE) in the predicted vs. observed maternal and fetal plasma concentration-time data (**Eq. 2**).

$AAFE= {10}^{\frac{1}{n}\sum|log\frac{predicted}{observed}|}$ (2)

Optimization of Feto-Placental ACS Pharmacokinetic Parameters (Including CL_int,Pgp,placenta_ ) (Figure 1, 3^rd^ panel)

Several factors determine fetal K_p,uu_ of the drug, that is the fetal:maternal unbound drug plasma concentration ratio at steady-state or the corresponding AUC ratio after a single dose or during the dosing interval at steady-state. fetal K_p,uu_ is determined by the drug’s transplacental clearance (i.e. intrinsic passive diffusion [CL_int,PD,placenta_], intrinsic P-gp-mediated efflux clearance [CL_int,Pgp,placenta_], intrinsic clearance via other placental transporters), placental metabolic clearance, placental blood flow, fetal hepatic intrinsic clearance. Our m-f PBPK model is populated with gestational age-dependent dynamic changes in fetal-maternal physiology. Since we do not have an estimate of the CL_int,Pgp,placenta_ of the ACS, we optimized its value to best explain the observed fetal-maternal ACS plasma concentration-time profiles. To do so, we first estimated *CL_int,PD,placenta_* and fetal hepatic intrinsic clearance and then estimated the K_p,uu_ of the drugs (**Figure 1**):

1. *Intrinsic Transplacental Passive Diffusion Clearance (CL_int,PD,placenta_) of the ACS*: The CL_int,PD,placenta_ for both drugs was determined as we have previously described [19]. Briefly, first we obtained the ratio of the apparent permeability of DEX and BET through Caco-2 cell monolayer (P_app, DEX/BET_ = 11.65x10^-6^ cm/s) relative to that of midazolam. Then, the *in vivo* CL_int,PD,placenta_ of midazolam (500 L/h) was scaled using this ratio to arrive at the CL_int,PD,placenta_ of the drugs. The estimated DEX/BET CL_int,PD,placenta_ of 118.9 L/h was higher than the placental blood flow at term (~45 L/h). Hence, the m-f PBPK model apparent CL_int,PD,placenta_ value for the ACS was set to 45 L/h (i.e. perfusion-limited transplacental clearance).
2. *Fetal Hepatic Intrinsic Clearance of the ACS:* Fetal hepatic intrinsic clearance was conservatively estimated from total intrinsic clearance in human liver microsomes phenotyping study (unpublished data) and scaling this value with total protein content per gram of liver tissue (26 mg) and the weight of fetal liver (~130 g) [19, 20]. Fetal hepatic intrinsic clearance of DEX/BET scaled to 1.18 L/h. This value is much smaller than the estimated placental intrinsic passive diffusion clearance of these drugs (45 L/h) and is therefore not expected to affect fetal exposure to the ACS [8]. Though fetal livers express CYP3A7 while adult livers express CYP3A4/5, our estimate is a conservative as CYP3A7 turnover of substrates tends to be lower than that by CYP3A4/5 [21, 22].
3. Optimization *of K_p,uu_* through sensitivity analysis*:* For BET, maternal and fetal plasma concentration-time profiles were simulated with various magnitudes of CL_int,Pgp,placenta_ until the predicted UV/MP ratio best described the observed data (by minimizing AAFE). CL_int,Pgp,placenta_ was expressed as a fraction of CL_int,PD,placenta_ (**Eq. 1**)*_._*

For this optimization, we used UV/MP ratio rather than the actual fetal plasma concentration-time profiles because the UV/MP ratios is less confounded by the observed large inter-individual variability in maternal and fetal plasma concentrations (note these values, each obtained at a single time point, are derived from many maternal-fetal pairs).The observed BET data (up to 96 h) [15] allowed us to determine the value of UV/MP ratio plateau, which reflects pseudo-equillibrium between maternal and fetal plasma concentrations. For DEX, this plateau value was not observed within the short sampling duration (6.5 h). Hence, for DEX, we estimated the “theoretical” value of this plateau by fitting a simple Emax model to the observed data using non-linear regression. Then, CL_int,Pgp,placenta_ was adjusted within the m-f PBPK model as described above until the simulated UV/MP ratio plateau achieved the Emax model-derived plateau.

Designing Alternative IM ACS Dosing Regimens by Predicting their Maternal-fetal Expsoure using the m-f PBPK model at GW30 (Figure 1, bottom panel)

Once the m-f PBPK model parameters for IM DEX-P and BET-P:A were optimized, they were adjusted for gestational week to GW30 (the median gestational age when these drugs are administered) and used to design alternative IM dosing regimens for these ACS that fulfill the following criteria:

1) To maintain fetal AUC or 5^th^ percentile C_min_ as follows:

1. For DEX, fetal drug exposure (AUC_0-48_) should be no less than that obtained after the reference DEX dosing regimen (6 mg q 12 h for 48 h) [23].
2. For BET, the fetal 5^th^ percentile C_min_ must be maintained above 1 ng/mL for 48h. Briefly, the downward adjustment of the dose was carried out until the predicted fetal 5^th^ percentile C_min_ reached 1ng/mL. The cutoff value of 1ng/ml is based on sheep data, where maintaining BET fetal plasma concentrations >1 ng/mL, over 36-48 h after drug administration, was necessary for fetal lung maturation [24, 25].

2) To not exceed maternal AUC or 95^th^ percentile C_max_ (or both) of the reference dosing regimen. Alternatively stated, provided the maternal AUC of the reference regimen is maintained, maternal 95^th^ percentile C_max_ may be exceeded.

3) The ACS dosing regimen must be convenient to administer (i.e., administer the ACS no more frequently than every 12 h).

**Table S1.** Non-pregnant PBPK model input parameters for DEX and BET

| **Parameter** | **DEX** | **BET** |
| --- | --- | --- |
| **Physico-chemical properties** |  |  |
| MW [g/mol] | 392.5 ^1^ | 392.5 ^1^ |
| Log P | 1.83 ^1^ | 1.83 ^1^ |
| Compound type | Monoprotic Base ^1^ | Monoprotic Base ^1^ |
| pK_a_ | -3.3 (basic) ^1^ 12.4 (acidic) ^1^ | -3.3 (basic) ^1^ 12.4 (acidic) ^1^ |
| f_u_ | 0.319 ^1^ | 0.36 ^1^ |
| B/P | 0.93 ^1^ | 1.12 ^1^ |
| **Absorption** |  |  |
| f_a_ | 1 (assumed) ^1^ | 1 (assumed) ^1^ |
| k_a_ [1/h] | 2.0 (IM) ^1^ | 2.0 (IV) ^1^ 1.5 (IM BET-P) ^3^ |
| T_lag_ [h] | 1 (IM) ^1^ | 1.5 (IM BET-P) ^3^ |
| **Distribution** |  |  |
| PBPK model | full ^1^ | full ^1^ |
| V_ss_ [L/kg] | 0.72 ^2^ | 1.3 ^1^ |
| K_p_ scalar | 0.22 ^1^ | 0.25 ^1^ |
| **Elimination** |  |  |
| CYP3A4 | CL_int_: 19 ^1^ | CL_int_: 8.3 ^1^ |
| CL_R_ [L/h] | 0.41 ^1^ | 0.49 ^1^ |
| Additional CL | 0.49 ^1^ [µL/min/mg] |  |

^1^ All the parameters are as published in Ke and Milad, 2019

^2^ DEX V_ss_ was as determined by Tsuei *et al.*, 1979

^3^ Optimized by us

*Note: SimCYP workspace, drug and population files can be provided upon request

Table S1 abbreviations and units

B/P - blood:plasma partition ratio; CL_int_ - intrinsic clearance [µL/min/mg protein]; CL_R_ - renal clearance [L/h]; CYP - cytochrome P450; f_a_ - fraction absorbed; f_u_ - fraction of unbound drug in plasma; HLM - human liver microsomes; k_a_ - absorption rate constant [h^-1^]; K_p_ - partition coefficient; log P - logarithm of octanol-water partition coefficient; MW - molecular weight [g/mol]; pK_a_ - negative decadal logarithm of acid dissociation constant; T_lag_ - absorption lag time [h]; pred - predicted; V_max_ - maximum rate of metabolite formation [pmol/min/mg microsomal protein]; V_ss_ - volume of distribution at steady state [L/kg]

Table S1 references

^1^ Ke, A. B., & Milad, M. A. (2019). Evaluation of Maternal Drug Exposure Following the Administration of Antenatal Corticosteroids During Late Pregnancy Using Physiologically-Based Pharmacokinetic Modeling. Clin Pharmacol Ther, 106(1), 164-173. doi:10.1002/cpt.1438

^2^ Tsuei, S. E., Moore, R. G., Ashley, J. J., & McBride, W. G. (1979). Disposition of synethetic glucocorticoids. I. Pharmacokinetics of dexamethasone in healthy adults. J Pharmacokinet Biopharm, 7(3), 249-264

**Figure S1**


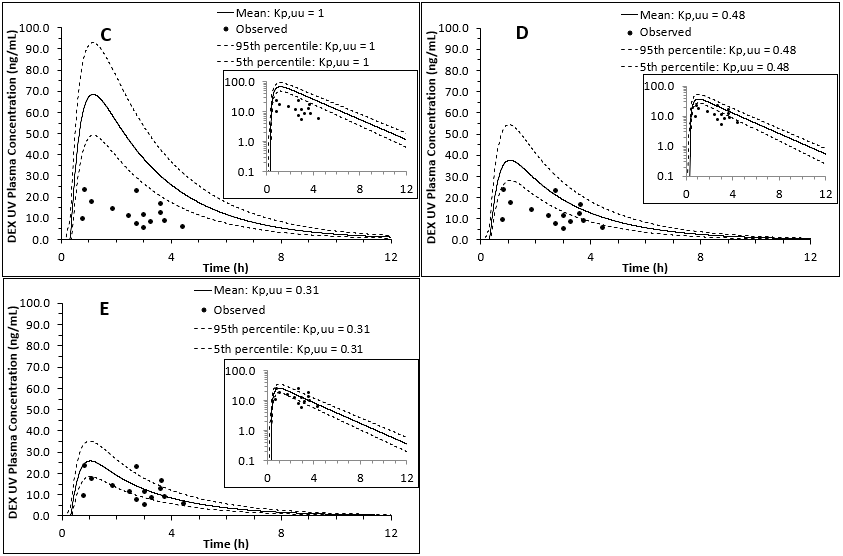

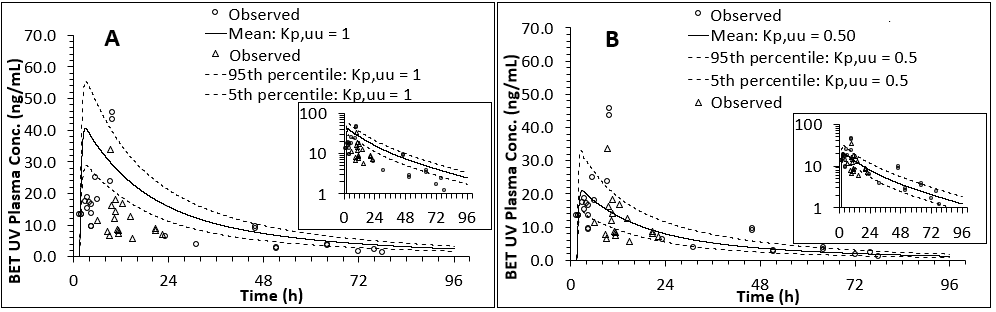


**Figure S1. Optimization of fetal plasma concentrations generated by our m-f PBPK model after intramuscular administration of BET-P:A (A-B) or DEX-P (C-E) to Caucasian pregnant women. (A-B)** Predicted mean BET UV C-T profiles (solid lines) and 5th and 95^th^ percentile (dashed lines) without (A: K_p,uu_=1) and with (B: K_p,uu_=0.50) P-gp efflux transport incorporated into the model. Observed data (empty triangles and circles) are from Ballabh *et al*., 2002 [15] and Foissac *et al.*, 2020 [16], respectively. **(C-E)** Predicted mean DEX UV C-T profiles (solid lines) and 5th and 99^th^ percentiles (dotted lines) without (C: K_p,uu_=1) and with (D: K_p,uu_=0.48 or E: K_p,uu_=0.31) Pgp efflux transport incorporated into the model. Observed data (filled circles) are from Tsuei *et al.*, 1980 [14].

**Figure S2**

**BET Alternative Regimen (2 mg IM BET-P q 12h, over 48 h,** **total 8 mg)**


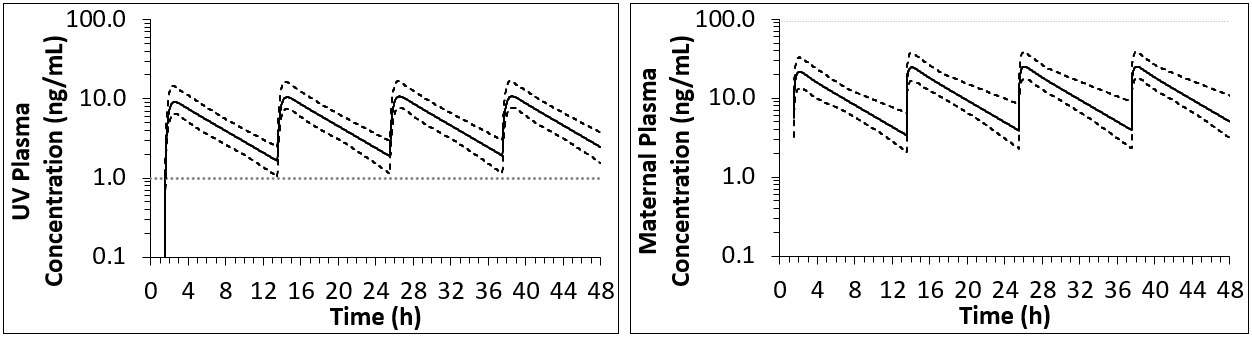


**Figure S2. Predicted fetal and maternal UV C-T profiles for the IM BET-P regimen using our m-f PBPK model.** This dosing regimen of IM BET-P resulted in fetal AUC_0-48_ of 248 ng*h/mL which is 66% lower than AUC_0-48_ of BET P:A reference regimen (724 ng*h/mL). Fetal UV 5^th^ percentile C_min_ remained above 1 ng/mL for the duration of drug administration (48 hr). Maternal AUC_0-48_ (542 ng*h/mL) and 95^th^ percentile C_max_ (38.2 ng/mL) were respective 38% and 41% of the BET-P:A reference regimen). Predicted mean C-T profiles are solid lines, 5^th^ and 95^th^ percentiles are dashed lines. Horizontal dotted lines in maternal C-T profiles denote maximum targeted cut-off value for maternal 95^th^ percentile C_max_ (94 ng/mL defined by the reference dosing regimen). Horizontal dotted lines in fetal C-T profiles denote minimum targeted cut-off value for fetal 5^th^ percentile C_min_ (1 ng/mL).

**Table S2. List of Abbreviations**

| **ABBREVIATION** | **DEFINITION** |
| --- | --- |
| 5^th^ percentile | 5th percentile confidence values |
| 95^th^ percentile | 95th percentile confidence values |
| ACS | Antenatal corticosteroids |
| AAFE | Absolute average fold error |
| AUC_f_ | Area under the curve of total fetal plasma concentration-time profile |
| AUC_m_ | Area under the curve of total maternal plasma concentration-time profile |
| BCRP | Breast cancer resistance protein |
| BET | Betamethasone |
| BET-A | Betamethasone acetate |
| BET-P | Betamethasone phosphate |
| BET-P:A | 1:1 betamethasone phosphate and acetate formulation |
| CI | Confidence interval |
| CL_int,PD,placenta_ | Intrinsic placental passive diffusion clearance |
| CL_hep,int_ | Intrinsic hepatic clearance |
| CL_int,Pgp,placenta_ | *In vivo* P-gp mediated efflux clearance from the placenta |
| C_max_ | Maximum plasma drug concentration |
| C_min_ | Minimum plasma drug concentration |
| CYP | Cytochrome P450 |
| DEX | Dexamethasone |
| DEX-P | Dexamethasone phosphate |
| E_max_ | Maximum effect |
| fCL_int_ | fetal Intrinsic hepatic clearance |
| f_u,f_ | Unbound fraction in fetal plasma |
| f_u,m_ | Unbound fraction in maternal plasma |
| GW | Gestational week |
| IM | Intramuscular |
| IV | Intravenous |
| k_a_ | Absorption rate constant |
| K_p_ | Partition coefficient |
| K_p,uu_ | Unbound partition coefficient |
| m-f PBPK model | Maternal-fetal physiologically based pharmacokinetic model |
| MDCK | Madin-Darby canine kidney |
| MP | Maternal plasma |
| P-gp | P-glycoprotein |
| RDS | Respiratory distress syndrome |
| T_lag_ | Absorption lag time |
| UV | Umbilical vein |
| V_ss_ | Volume of distribution at steady-state |

**SUPPLEMENTARY INFORMATION REFERENCES:**

1. Ke, A.B. and M.A. Milad, *Evaluation of Maternal Drug Exposure Following the Administration of Antenatal Corticosteroids During Late Pregnancy Using Physiologically-Based Pharmacokinetic Modeling.* Clin Pharmacol Ther, 2019. **106**(1): p. 164-173.

2. Tsuei, S.E., et al., *Disposition of synethetic glucocorticoids. I. Pharmacokinetics of dexamethasone in healthy adults.* J Pharmacokinet Biopharm, 1979. **7**(3): p. 249-64.

3. Petersen, M.C., et al., *Pharmacokinetics of betamethasone in healthy adults after intravenous administration.* Eur J Clin Pharmacol, 1983. **25**(5): p. 643-50.

4. Jobe, A.H., et al., *Pharmacokinetics and Pharmacodynamics of Intramuscular and Oral Betamethasone and Dexamethasone in Reproductive Age Women in India.* Clin Transl Sci, 2020. **13**(2): p. 391-399.

5. Varis, T., et al., *The cytochrome P450 3A4 inhibitor itraconazole markedly increases the plasma concentrations of dexamethasone and enhances its adrenal-suppressant effect.* Clin Pharmacol Ther, 2000. **68**(5): p. 487-94.

6. Petersen, M.C., et al., *Disposition of betamethasone in parturient women after intravenous administration.* Eur J Clin Pharmacol, 1983. **25**(6): p. 803-10.

7. Peets, E.A., M. Staub, and S. Symchowicz, *Plasma binding of betamethasone-3H, dexamethasone-3H, and cortisol-14C--a comparative study.* Biochem Pharmacol, 1969. **18**(7): p. 1655-63.

8. Zhang, Z., et al., *Development of a Novel Maternal-Fetal Physiologically Based Pharmacokinetic Model I: Insights into Factors that Determine Fetal Drug Exposure through Simulations and Sensitivity Analyses.* Drug Metab Dispos, 2017. **45**(8): p. 920-938.

9. Zhang, Z., et al., *Prediction of gestational age-dependent induction of in vivo hepatic CYP3A activity based on HepaRG cells and human hepatocytes.* Drug Metab Dispos, 2015. **43**(6): p. 836-42.

10. Hebert, M.F., et al., *Effects of pregnancy on CYP3A and P-glycoprotein activities as measured by disposition of midazolam and digoxin: a University of Washington specialized center of research study.* Clin Pharmacol Ther, 2008. **84**(2): p. 248-53.

11. De Sousa Mendes, M., et al., *A Physiologically-Based Pharmacokinetic Model to Predict Human Fetal Exposure for a Drug Metabolized by Several CYP450 Pathways.* Clin Pharmacokinet, 2017. **56**(5): p. 537-550.

12. Xia, B., et al., *A simplified PBPK modeling approach for prediction of pharmacokinetics of four primarily renally excreted and CYP3A metabolized compounds during pregnancy.* AAPS J, 2013. **15**(4): p. 1012-24.

13. Dallmann, A., et al., *A Physiologically Based Pharmacokinetic Model for Pregnant Women to Predict the Pharmacokinetics of Drugs Metabolized Via Several Enzymatic Pathways.* Clin Pharmacokinet, 2018. **57**(6): p. 749-768.

14. Tsuei, S.E., et al., *Disporition of synthetic glucocorticoids. II. Dexamethasone in parturient women.* Clin Pharmacol Ther, 1980. **28**(1): p. 88-98.

15. Ballabh, P., et al., *Pharmacokinetics of betamethasone in twin and singleton pregnancy.* Clin Pharmacol Ther, 2002. **71**(1): p. 39-45.

16. Foissac, F., et al., *Maternal Betamethasone for Prevention of Respiratory Distress Syndrome in Neonates: Population Pharmacokinetic and Pharmacodynamic Approach.* Clin Pharmacol Ther, 2020. **108**(5): p. 1026-1035.

17. Ballard, P.L., P. Granberg, and R.A. Ballard, *Glucocorticoid levels in maternal and cord serum after prenatal betamethasone therapy to prevent respiratory distress syndrome.* J Clin Invest, 1975. **56**(6): p. 1548-54.

18. Gyamfi, C., et al., *The effect of plurality and obesity on betamethasone concentrations in women at risk for preterm delivery.* Am J Obstet Gynecol, 2010. **203**(3): p. 219 e1-5.

19. Zhang, Z. and J.D. Unadkat, *Development of a Novel Maternal-Fetal Physiologically Based Pharmacokinetic Model II: Verification of the model for passive placental permeability drugs.* Drug Metab Dispos, 2017. **45**(8): p. 939-946.

20. Abduljalil, K., et al., *Anatomical, physiological and metabolic changes with gestational age during normal pregnancy: a database for parameters required in physiologically based pharmacokinetic modelling.* Clin Pharmacokinet, 2012. **51**(6): p. 365-96.

21. Williams, J.A., et al., *Comparative metabolic capabilities of CYP3A4, CYP3A5, and CYP3A7.* Drug Metab Dispos, 2002. **30**(8): p. 883-91.

22. Stevens, J.C., et al., *Developmental expression of the major human hepatic CYP3A enzymes.* J Pharmacol Exp Ther, 2003. **307**(2): p. 573-82.

23. Collaborators, W.A.T., et al., *Antenatal Dexamethasone for Early Preterm Birth in Low-Resource Countries.* N Engl J Med, 2020. **383**(26): p. 2514-2525.

24. Schmidt, A.F., et al., *Low-dose betamethasone-acetate for fetal lung maturation in preterm sheep.* Am J Obstet Gynecol, 2018. **218**(1): p. 132 e1-132 e9.

25. Kemp, M.W., et al., *The efficacy of antenatal steroid therapy is dependent on the duration of low-concentration fetal exposure: evidence from a sheep model of pregnancy.* Am J Obstet Gynecol, 2018. **219**(3): p. 301 e1-301 e16.
